# Inhibitory effect of exercise on elevated blood pressure and fetal growth restriction during pregnancy in Dahl salt‐sensitive rats

**DOI:** 10.14814/phy2.70298

**Published:** 2025-04-09

**Authors:** Toru Kobayashi, Linh Thuy Pham, Mutsumi Kobayashi, Ko Yamanaka, Atsuo Itakura, Hidefumi Waki

**Affiliations:** ^1^ Department of Obstetrics and Gynecology Juntendo University Tokyo Japan; ^2^ Department of Obstetrics and Gynecology, Graduate School of Medicine Juntendo University Tokyo Japan; ^3^ Department of Physiology, Graduate School of Health and Sports Science Juntendo University Chiba Japan; ^4^ Institute of Health and Sports Science & Medicine Juntendo University Chiba Japan

**Keywords:** Baroreflex, Dahl salt‐sensitive rat, Exercise, Hypertension, Rostral ventrolateral medulla

## Abstract

Exercise is effective in preventing gestational hypertension, but its mechanism remains unclear. This study investigates the effects of exercise on Dahl salt‐sensitive (DSS) rats, which develop elevated blood pressure and fetal growth restriction during pregnancy. DSS rats were divided into exercise and non‐exercise groups, with Sprague–Dawley rats as controls. Exercise consisted of voluntary running, starting 4 weeks prior to pregnancy until the last trimester. Cardiovascular parameters, molecular characteristics of the brain and placenta, and fetal conditions were evaluated. Exercise significantly improved elevated blood pressure at early pregnancy and was associated with improved baroreceptor reflex gain. Gene expression analysis in the rostral ventrolateral medulla (RVLM) showed exercise‐induced downregulation of nitric oxide synthase and upregulation of superoxide dismutase. These genetic changes suggest that exercise impacts circulatory regulation mechanisms, contributing to blood pressure improvement. In addition, placental analysis revealed a marked increase in placental growth factor expression due to exercise. In conclusion, exercise alleviates elevated blood pressure at early gestation and fetal growth restriction in DSS rats. Genetic modifications in the RVLM may play a critical role in exercise‐induced cardiovascular improvements. This study highlights the potential of exercise as a therapeutic approach for managing gestational elevated blood pressure and fetal growth restriction and provides insights into its underlying mechanisms.

## Graphical abstract

Exercise in pregnant Dahl salt‐sensitive rats leads to gene expression changes in the brain and placenta during the first trimester.

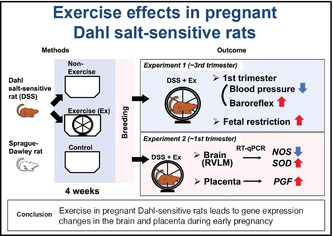

## 1 INTRODUCTION

Hypertension during pregnancy is a significant health concern for pregnant women (Say et al., 2014). Hypertensive disorders of pregnancy (HDP) markedly impact perinatal outcomes adversely for both the mother and child (Tooher et al., 2017). Despite its prevalence, options for treating and preventing hypertension during pregnancy remain limited (Chappell et al., 2021).

Several clinical and animal studies have shown that exercise prevents hypertension during pregnancy (Falcao et al., 2010; Liu et al., 2022; Sorensen et al., 2003; Witvrouwen et al., 2020). While clinical studies have proven the effects of exercise, animal studies are essential to identify causal pathways, develop new therapies, and evaluate safety and efficacy of interventions. Through animal studies, the mechanisms underlying the effects of exercise have been elucidated (Gatford et al., 2020).

Previous animal studies have highlighted the pathways through which exercise may attenuate hypertension symptoms. For instance, Gilbert et al. showed that exercise attenuated placental ischemia‐induced hypertension, angiogenic imbalance, and oxidative stress in reduced uteroplacental perfusion rats (Gilbert et al., 2012), while Falcao et al. demonstrated its effectiveness in reducing hypertension, proteinuria, and abnormal placental findings in transgenic mice overexpressing human angiotensinogen and renin (Falcao et al., 2010). However, exercise effects have been reported only in surgical (Gilbert et al., 2012) and genetic manipulation (Falcao et al., 2010) animal models, which differ from human pregnant women who spontaneously develop hypertension (Gatford et al., 2020).

Dahl salt‐sensitive (DSS) rats can effectively serve as human superimposed preeclampsia (PE) models because they originally had hypertension and urinary protein and spontaneously developed PE symptoms without salt during pregnancy (Gillis et al., 2015). However, the mechanisms underlying the effects of exercise in this model animal remain unclarified.

The mechanism underlying the effect of exercise on HDP may result from its ability to ameliorate oxidative stress and inflammatory cytokines, which are responsible for vascular endothelial damage (Genest et al., 2012). These have also been reported in general hypertension. In addition, the mechanism of the antihypertensive effect of exercise may be related to the circulatory regulation of the autonomic nervous system (Groehs et al., 2015; Waki et al., 2003). In particular, the baroreceptor areas of the rostral ventral lateral medulla (RVLM) and nucleus tractus solitarius (NTS) markedly contribute to the generation and maintenance of sympathetic nerve activity and are important parts of the central baroreflex pathway (Moraska et al., 2000). Thus, we hypothesize that autonomic circulatory regulation may be a critical mechanism underlying the suppression of blood pressure elevation during pregnancy.

To understand the mechanisms underlying exercise‐induced protection from HDP symptoms, we examined the effects of daily exercise in pregnant DSS rats on cardiovascular parameters, molecular biological characteristics of brain cardiovascular centers, and placenta. We also examined the potential mechanisms behind the enhanced fetal development in Dahl rats by daily exercise.

## 2 MATERIALS AND METHODS

### 2.1 Experiment 1

**FIGURE 1:**
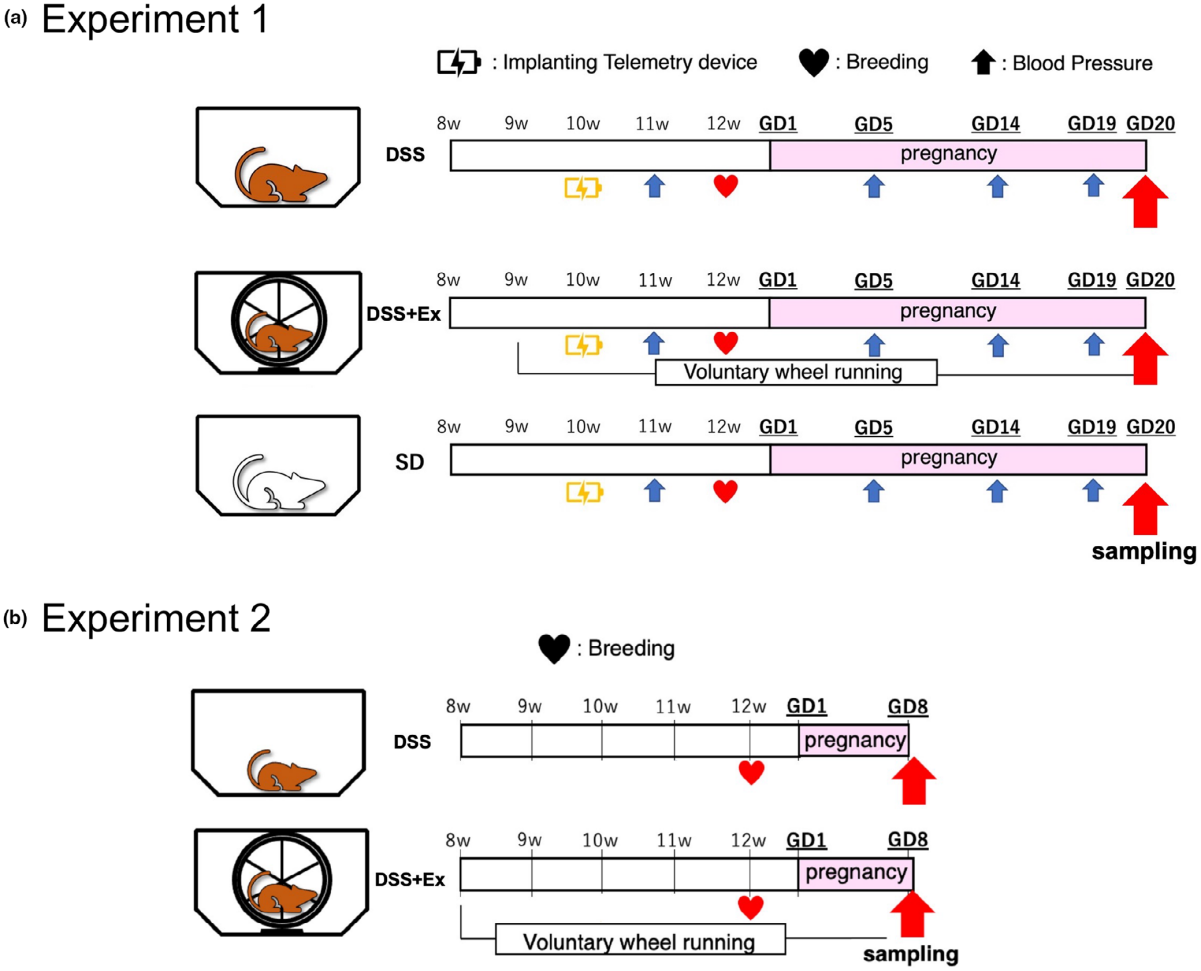
Experimental flows. (a) Female DSS rats were randomly divided into two groups: A non‐exercise group (DSS, *n* = 11) or an exercise group (DSS + Ex, *n* = 11). DSS + Ex rats were given access to a running wheel 4 weeks prior to breeding and throughout pregnancy. SD rats (*n* = 10) were housed without a running wheel, similar to the DSS group. (b) To investigate circulatory regulation in early pregnancy, brains and placentas were collected on gestational day 8 as Experiment 2. DSS rats were randomly divided into two groups: a non‐exercise group (DSS, *n* = 6) and an exercise group (DSS + Ex, *n* = 6).

DSS rats (DIS/Eis) and Sprague–Dawley (SD) rats were obtained from Japan SLC (Shizuoka, Japan). The animals were housed in a temperature‐controlled environment (room temperature: 24 ± 1°C, humidity: 55 ± 5%) under a fixed 12:12‐h dark/light cycle (6:00–18:00/18:00–6:00). Female and male rats were provided with ad libitum access to normal food (Rodent LabDiet EQ, 5L37, 1.1%NaCl, PMI Nutrition International, MO, USA) and water. In addition, 8‐week‐old female DSS rats were randomly divided into two groups: non‐exercise group (DSS) or exercise group (DSS + Ex), and SD rats served as controls. DSS + Ex rats were placed in cages with access to a running wheel 4 weeks prior to breeding and throughout pregnancy. Timed breeding was performed for each strain in a wire bottom cage, and the presence of vaginal plugs was indicative of gestational day (GD) 1. After breeding, the females were returned to individual cages with exercise wheels or without wheels until the morning of GD 20 (Figure 1a). Animals were euthanized by 5% isoflurane inhalation followed by a secondary physical method including bilateral thoracotomy and brain tissue harvest. This study was conducted following the guidelines of the Japan Physiological Society with the approval of the Animal Experiment Ethics Committee of Juntendo University (approval number: S22; 2021‐16, 2022‐19, 2023‐19) and performed in accordance with the National Institutes of Health (NIH) Guide for the Care and Use of Laboratory Animals (eighth edition, 2011).

### 2.2 Voluntary wheel exercise

Wheel rotations were measured using a data recording system (CIF‐4A, Melquest, Toyama, Japan). Voluntary wheel running was chosen for this study to minimize the potentially harmful effects previously reported with treadmill running in SD rats (Moraska et al., 2000). One full rotation of the running wheel corresponds to approximately 1 m.

### 2.3 Blood pressure

Rats were implanted with telemetry devices (HD‐S10; Data Sciences International, St. Paul, MN, USA) to measure mean arterial pressure (MAP) from the abdominal aorta, as previously reported (Waki et al., 2006; Yamanaka et al., 2018), at 10 weeks old. After 2 weeks, baseline points were measured prior to mating. MAP was recorded during early, mid, and late pregnancy (GD 4‐5, GD 13‐14, and GD19‐20, respectively).

### 2.4 Baroreflex and PI variability parameters

The cardiac vagal baroreflex is a mechanism that maintains blood pressure stability by adjusting the heart rate via the vagus nerve, responding to changes in blood pressure levels to either slow down or speed up the heart rate. Many cardiovascular diseases, including hypertension, are associated with a change in spontaneous baroreceptor reflex gain (sBRG) (La Rovere et al., 2001; Sakamoto et al., 2016). The sBRG was calculated from the pulsatile pressure wave using Hey‐Presto software (Waki et al., 2006). The sBRG was determined from the spontaneous changes in systolic blood pressure (SBP) and pulse interval (PI, the time interval between consecutive heartbeats, inversely related to heart rate), as previously reported. First, the moving averages of SBP and PI were calculated over 10 cardiac cycles. Second, from these moving average data, spontaneously occurring ramps of either decreasing or increasing SBP of ≥4 beats were used to calculate the BRG. Third, for each pair of SBP and PI ramps, measurements were performed at delays of three, four, and five beats. Finally, from these three ramps, plots of changes in PI and SBP were constructed to form slopes for each delay. The averaged slope values were calculated for each delay. The sBRG values represent the mean of the three measurements (Waki et al., 2006).

PI variability represents the beat‐to‐beat fluctuations in the intervals between consecutive heartbeats, reflecting autonomic nervous system activity. PI variability was analyzed using Hey‐Presto software. For each 10‐min measurement period, beat‐to‐beat PIs were converted into data points (every 100 ms) using a spline interpolation. The resulting time series were divided into half‐overlapping sequential sets of 512 data points. For each data set, after the removal of the linear trend and application of the Hanning window, power spectral density was computed using the Fast Fourier Transform (FFT) algorithm (Waki et al., 2006). Within PI variability analysis, low‐frequency (LF) power represents both sympathetic and parasympathetic activity, high‐frequency (HF) power reflects parasympathetic activity, and the LF/HF ratio reflects sympathetic activity (Pagani et al., 1986).

### 2.5 Urine protein

Urine samples were collected in a wire bottom cage for 30 min. This method avoids the unnecessary stress of placing the animals in metabolic cages for 24 h. Indeed, mice of either sex showed a significant increase in MAP and HR when placed in metabolic cages (Hoppe et al., 2009). Urine was frozen at −80°C for future analysis. Proteinuria was assessed by measuring the albumin/creatinine ratio (ACR mg/g) using a DCA 2000 immunoassay system (6010A, Siemens Healthcare Diagnostics, Tokyo, Japan) (Kim et al., 2015).

### 2.6 Tissue collection

Rats were anesthetized with isoflurane on GD8 (Experiment 2) or GD20 (Experiment 1) as described above. After blood collection from the left ventricle, the brain was rapidly removed from the cranium. The placenta and fetus were then collected and weighed individually only in Experiment 1.

### 2.7 Serum assays

The serum obtained after centrifugation was immediately frozen and stored at −80°C. The antiangiogenic soluble vascular endothelial growth factor (VEGF) receptor 1 (sFlt1) is one of the markers of PE, because it is found at much higher circulating levels in women with active PE and inactivates angiogenic factors such as VEGF and placental growth factor (PGF). Circulating sFlt‐1 concentrations were measured using commercial enzyme‐linked immunosorbent assay kits available from R&D systems (catalog MVR100, Quantikine®; Minneapolis, MN).

### 2.8 RNA sequencing

Total RNAs were extracted from the placenta with Invitrogen™ TRIzol reagent (15596026, Thermo Fisher Scientific, Waltham, MA, USA) and the RNeasy Mini Kit with on‐column DNase treatment (74104 QIAGEN, Hilden, Germany). RNA quality was confirmed using Bioanalyzer and RNA‐Seq libraries prepared using the NEBNext® UltraTMII Directional RNA Library Prep Kit according to the Illumina protocol. Multiplexed libraries were validated using Bioanalyzer, normalized, and pooled for sequencing. High‐throughput sequencing was performed on the NovaSeq 6000 system (Illumina, california, USA) with a 150‐bp read length.

### 2.9 Experiment 2

Since the results of Experiment 1 indicated that elevated blood pressure in DSS rats occurred in early pregnancy, Experiment 2 was conducted in early pregnancy to confirm the effect of exercise on autonomic circulatory regulation (Figure 1b).

Here, 8‐week‐old female DSS rats (DIS/Eis) (Japan SLC, Shizuoka, Japan) were randomly divided into two groups: a non‐exercise group (DSS; *n* = 6) or an exercise group (DSS + Ex; *n* = 6). Breeding conditions and mating methods were the same as in Experiment 1, and tissue collection was performed on GD8. This is because the placenta becomes visible to the naked eye on GD8. On GD8, the NTS and RVLM of the brain and placenta were collected, mRNA was extracted, and gene expression analysis was performed. Blood and urine samples were also collected. The study was conducted following the guidelines of the Japan Physiological Society, with the approval of the Animal Experiment Ethics Committee of Juntendo University (approval number: S46; 2023‐23).

### 2.10 Tissue collection and RNA extraction

The brain was rapidly removed from the cranium, after which the blocks of the dorsal part of the medulla oblongata and the forebrain were isolated and placed on an ice‐cold platform. The NTS located between 1.0 mm rostral and 0.5 mm caudal to the calamus scriptorius (CS) was dissected under a dissecting microscope (Onishi et al., 2018). To collect the RVLM, the medulla oblongata is first divided into two sections in the sagittal plane and then further divided vertically at the level of the CS. It is located 2 mm rostral to this section and 2 mm ventral to the lateral 2 mm. A rat brain stereotaxic atlas was used as a reference (Paxinos and Watson, 2013). However, we could not exclude the possibility that some cells from the surrounding tissues may have contaminated the NTS and RVLM samples during the dissection process. The tissues were immediately homogenized in Invitrogen™ TRIzol reagent (15596026, Thermo Fisher Scientific, Waltham, MA, USA) and stored at −80°C before being processed. A single operator performed all dissections. Total RNA from each sample was isolated according to the protocol supplied by the manufacturer and was stored at −80°C. The purity (260/230 nm and 260/280 nm ratios of 1.7) and integrity (RNA integrity number of 7) of all total RNA samples were verified using a NanoDrop ONE spectrophotometer (Thermo Fisher Scientific, Waltham, MA, USA), respectively.

### 2.11 Reference gene stability

We first established a stable reference gene in the NTS, RVLM, and placenta of the two groups, as previously reported (Nguyen et al., 2023). The expression levels of 11 rat housekeeping genes, including those of actin beta (Actb), beta‐2 microglobulin (B2m), peptidylprolylisomerase (cyclophylin A) (Ppia), transferrin receptor (Tfrc), ribosomal protein large P1 (Rplp1), hypoxanthine phosphoribosyl transferase 1 (Hprt1), lactate dehydrogenase (Ldha), phosphoglycerate kinase 1 (Pgk1), TATA box binding protein (Tbp), non‐POU domain‐containing octamer‐binding (Nono), and succinate dehydrogenase complex, subunit A, flavoprotein (Sdha), were compared between the three groups (Table 1). The One‐Step TB Green Prime Script PLUS RT‐PCR Kit (RP096B, Takara Bio, Shiga, Japan) with Rat Perfect Real‐Time Primers (RR036A, Takara Bio, Shiga, Japan) was used on a CronoSTAR™ 96 Real‐Time PCR System (640231, Takara Bio, Shiga, Japan), and the raw cycle threshold (Ct) values were collected. All samples were run in duplicate. To determine reference gene stability, we uploaded the 2^−ΔΔCT^ values of each sample to RefFinder (Xie et al., 2023). RefFinder is a user‐friendly, web‐based, comprehensive tool developed for evaluating and screening reference genes from extensive experimental datasets. It integrates the currently available major computational programs (geNorm, Normfinder, BestKeeper, and the comparative Δ‐Ct method) to compare and rank the tested candidate reference genes. Based on the rankings from each program, it assigns an appropriate weight to an individual gene and calculates the geometric mean of their weights for the overall final ranking.

**TABLE 1:** qPCR primer sequences used for gene expression analyses.

Gene	GenBank accesion No.	Gene size (bp)	Forward	Reverse
Actb	NM_031144.3	1293	GGAGATTACTGCCCTGGCTCCTA	GACTCATCGTACTCCTGCTTGCTG
B2m	NM_012512.2	1845	CCTGGCTCACACTGAATTCACAC	AACCGGATCTGGAGTTAAACTGGTC
Casp3	NM_012922.2	2484	GCAGCAGCCTCAAATTGTTGAC	TGCTCCGGCTCAAACCATC
Cybb	NM_023965.1	4248	GCTCAACCAGAATTCGAAGACAAC	GTCCCGACTCTGGCATTCAC
Hif1a	NM_024359.2	4003	TCTAGTGAACAGGATGGAATGGAG	TCGTAACTGGTCAGCTGTGGTAA
Hprt1	NM_012583.2	1260	TCCTCATGGACTGATTATGGACA	TAATCCAGCAGGTCAGCAAAGA
Nfkb1	NM_001276711.1	4062	CACCTCTACACATAGCAGCTGGAA	CCCAAGAGTCGTCCAGGTCATA
Nono	NM_001012356.1	2438	GCCAGATGGAACCCTTGGA	GCGGCGTTTATTTGGAGCA
Nos1	NM_052799.1	4594	TCAAAGCCATCCAGCGCATA	ACGTTCTGAGGGTGACTCCAAAG
Nos2	NM_012611.3	3793	CAAACTGTGTGCCTGGAGGTTC	AAGTAGGTGAGGGCTTGCCTGA
Nox1	NM_053683.2	2571	ACAAATTCCAGCGTGCACACA	CGGTTTGCCTAATTCGTCCATC
Pgf	NM_053595.2	1586	CTGCTGGGAACAACTCAACAGAA	TCTCCATTGGCCGGCAGTA
Pgk1	NM_053291.3	1685	TCCATGGTGGGTGTGAATCTG	CAGCTGGATCTTGTCTGCAACTTTA
Ppia	NM_017101.1	743	GGCAAATGCTGGACCAAACAC	AAACGCTCCATGGCTTCCAC
Sdha	NM_130428.1	2277	CTTCAGCTGTACTTCTGCCCACAC	TGAGGCAGCCAGCACCATAG
Sod1	NM_017050.1	650	CATGGGTTCCATGTCCATCAATA	AGCCACATTGCCCAGGTCTC
Sod2	NM_017051.2	2090	GGTGTGAGCTGCTCTTGATTGA	TTGATGGCCTTATGATGACAGTGA
Tbp	NM_001004198.1	1923	GCTGCAGTCATCATGAGAATAAGAG	CACCATGTTCTGGATCTTGAAGT
Tfrc	NM_022712.1	4805	GATGGATCAAGCCAGATCAGCA	GCAGCCAGTTTCATCTCCACA
Tnf	NM_012675.3	1687	CTCCGGGCTCAGAATTTCCA	ATCGACATTCCGGGATCCAG
Tnf	NM_012675.3	1687	CTCCGGGCTCAGAATTTCCA	ATCGACATTCCGGGATCCAG
Vegfa	NM_031836.2	3546	GCACGTTGGCTCACTTCCAG	TGGTCGGAACCAGAATCTTTATCTC

### 2.12 RT‐qPCR

Genomic DNA was depleted using Invitrogen™ amplification‐grade DNase I (18068015 Invitrogen, Thermo Fisher Scientific, USA). The One‐Step TB Green Prime Script PLUS RT‐PCR Kit (RP096B, Takara Bio, Shiga, Japan) with Rat Perfect Real‐Time Primers (RR036A, Takara Bio, Shiga, Japan) was used on a CronoSTAR™ 96 Real‐Time PCR System (640231, Takara Bio, Shiga, Japan), and the raw cycle threshold (Ct) values were collected. The fold‐change (FC) values were determined by the 2^−ΔΔCt^ method (Witvrouwen et al., 2020), and the raw Ct values were normalized to the mean of the most stable housekeeping genes. Table 1 contains the sequences of custom‐designed primers. Primers were selected based on previous studies for oxidative stress, pro‐inflammatory agents, and neovascular factors (Ahmed et al., 2000; Farah et al., 2021; Li et al., 2018).

### 2.13 Statistical analysis

All data are presented as mean ± SD. The mean values of the two groups were compared using Student's *t*‐test for parametric results and Mann–Whitney *U* (MWU) test for nonparametric results. Results obtained over time were analyzed using repeated two‐way ANOVA with Bonferroni post hoc test, and differences in the mean values of more than three groups were statistically analyzed using one‐way ANOVA and Tukey's post hoc test. Statistical analyses were performed with EZR (Saitama Medical Center, Jichi Medical University, Saitama, Japan), which is a graphical user interface for R (The R Foundation for Statistical Computing, Vienna, Austria) (Kanda, 2013).

## 3 RESULTS

### 3.1 Experiment 1

#### 3.1.1 Voluntary wheel exercise

**FIGURE 2:**
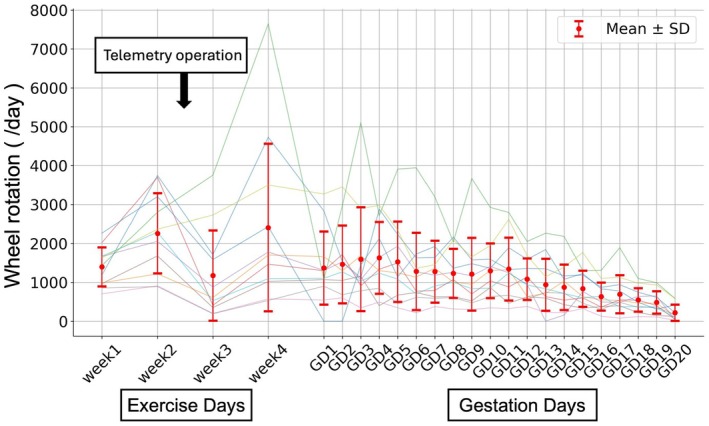
Daily wheel rotation before and during pregnancy in DSS + Ex. Up until Week 4, the data represent the average running distance per week. After Week 2, telemetry surgery was performed. Telemetry could not be measured during any period in five out of the 11 DSS rats. The red plots and lines indicate the mean ± SD (*n* = 6).

The physical activity of DSS (*n* = 11) rats is shown in Figure 2. It is demonstrated that as gestational age progresses towards the late stages, physical activity decreases. Additionally, as shown in Figure 2, there is a notable degree of individual variability.

#### 3.1.2 Blood pressure

**FIGURE 3:**
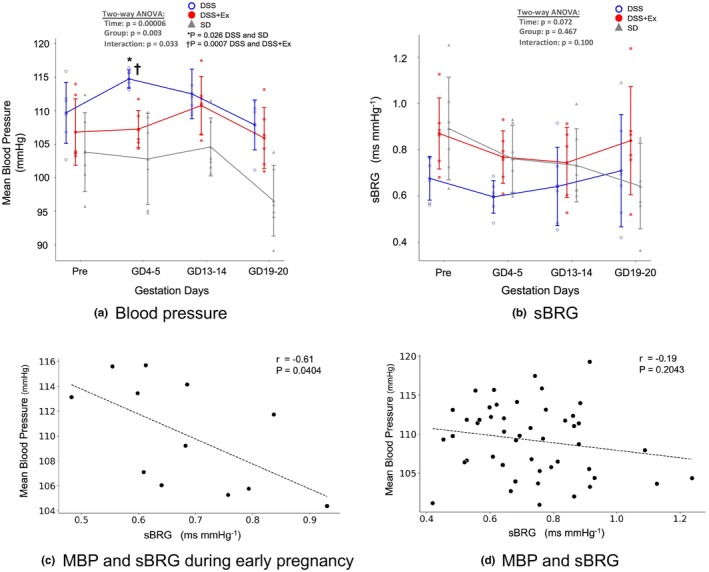
Effect of exercise on mean blood pressure and spontaneous baroreceptor reflex gain. (a) MAP changes in DSS (*n* = 6), DSS + Ex (*n* = 6), and SD (*n* = 6) groups. On GD4–5, the MAP of DSS markedly surpasses those of DSS + Ex and SD (Time: *p* < 0.001, Group: 0.003, Interaction: *p* = 0.033). (b) Spontaneous baroreceptor reflex gain (sBRG). The repeated measures two‐way ANOVA did not reveal a significant interaction effect between group and time for sBRG (Time: *p* = 0.072, Group: 0.46, Interaction: *p* = 0.100). (c) Correlation between MAP and sBRG during early pregnancy (GD4–5) in DSS, DSS + Ex. A significant negative correlation was observed (*r* = −0.61, *p* = 0.0404). (d) Correlation between MAP and sBRG across the entire pregnancy in DSS, DSS + Ex. No significant correlation was observed (*r* = −0.19, *p* = 0.2043). Data are presented as mean ± SD and analyzed using one‐way or two‐way ANOVA where applicable and Pearson's correlation analysis.

MAP before gestation was similar across all groups (DSS: 109.6 ± 1.9 mmHg, DSS + Ex: 106.8 ± 2.0 mmHg, and SD: 103.8 ± 2.4 mmHg). On early pregnancy (GD4–5), MAP increased significantly in the DSS group (114.7 ± 0.5 mmHg), whereas exercise prevented an increase in MAP (107.2 ± 1.1 mmHg). On mid and late pregnancy, MAP values were not different between the DSS and DSS + Ex groups (Figure 3a). Notably, there were 4–5 rats in which the telemetry devices were implanted but either did not function properly or failed to provide accurate measurements (not function or failed; DSS: 5, DSS + Ex: 5, SD: 4).

#### 3.1.3 BRG and PI variability parameters

A repeated two‐way ANOVA showed no significant differences in spontaneous baroreceptor reflex gain (sBRG) among the three groups (*p* = 0.10), nor any interaction with time (*p* = 0.07). (Figure 3b). Correlation analysis in DSS and DSS + Ex groups revealed that on early pregnancy (GD4–5), there was a significant negative correlation between sBRG and MAP (*r* = −0.61, *p* = 0.0404; Figure 3c). However, when data from the entire pregnancy were analyzed, no significant correlation was observed between sBRG and MAP (*r* = −0.19, *p* = 0.2043; Figure 3d).

**FIGURE 4:**
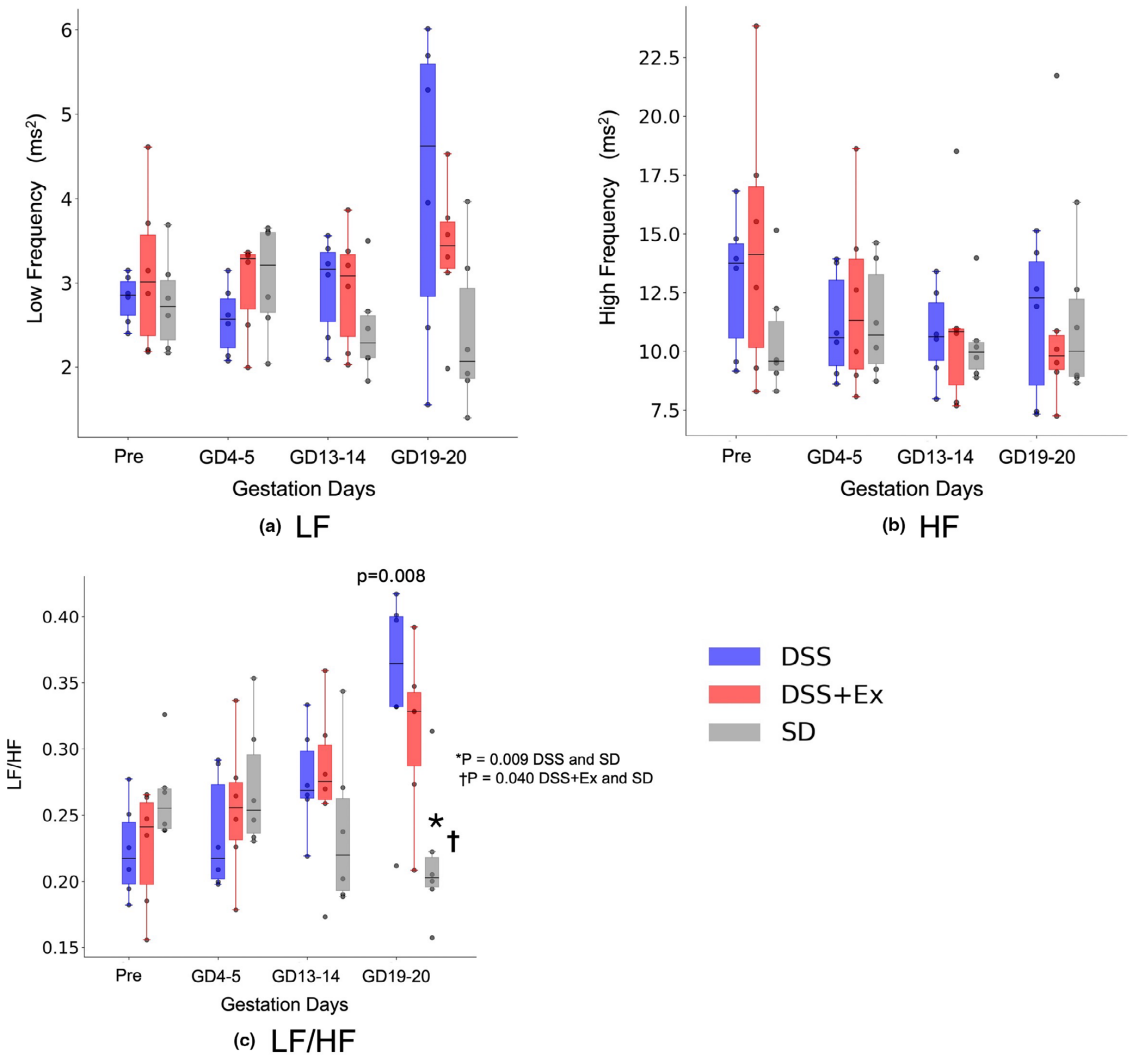
Effect of exercise on pulse interval variability parameters. Changes in PI variability parameters during pregnancy in DSS, DSS + Ex, and SD groups. (a) Low‐frequency (LF, ms^2^). (b) High‐frequency (HF, ms^2^). (c) LF/HF ratio. No significant differences were observed in LF or HF across the groups at any time point. For LF/HF, significant differences were found in late gestation (GD19‐20), where SD was significantly lower than DSS (*p* = 0.009) and DSS + Ex (*p* = 0.040). Data are presented as mean ± SD and analyzed using one‐way ANOVA.

No significant differences were observed in pulse interval (PI) variability parameters, including low‐frequency (LF), high‐frequency (HF), and the LF/HF ratio, across all gestational periods between DSS and DSS + Ex (Figure 4).

#### 3.1.4 Fetus growth

**FIGURE 5:**
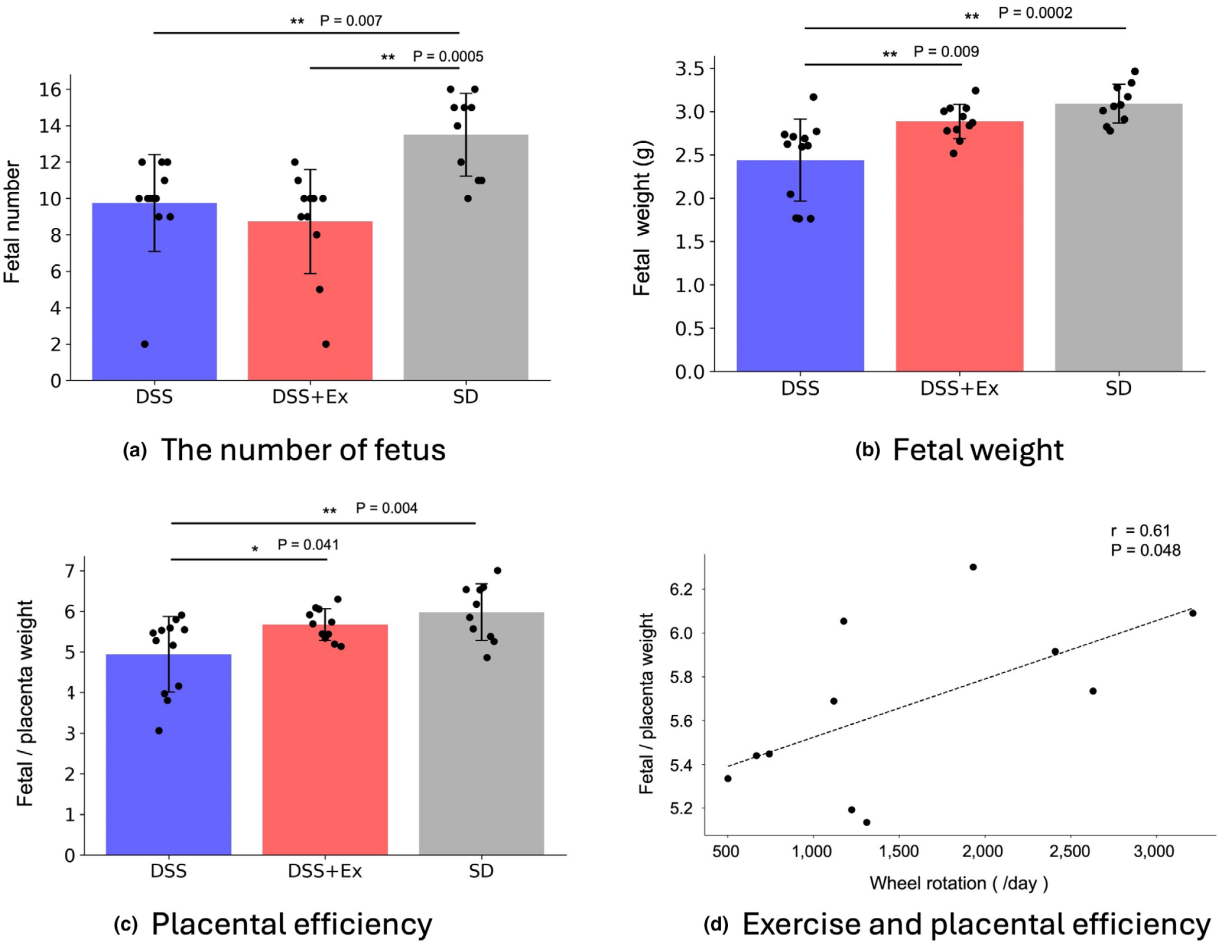
Effects of exercise on fetal outcomes. Fetal number (a), fetal weight (b), placental efficiency (c), and correlation between exercise and placental efficiency in DSS + Ex (d). Significant differences were observed among groups in a (*p* = 0.0006), b (*p* = 0.0002), and c (*p* = 0.006). These parameters were compared in the DSS (*n* = 11), DSS + Ex (*n* = 11), and SD (*n* = 10) groups. Data were expressed as mean ± SD and analyzed by one‐way ANOVA and Pearson's correlation analysis.

SD rats presented a markedly higher fetus number than non‐exercise and exercise DSS rats (Figure 5a). Similar to previous studies, SD rats had significantly higher fetal weights than the DSS group. (Gillis et al., 2015) In contrast, the DSS + Ex group gained enough weight to equal that of the SD group (Figure 5b). Furthermore, placental efficiency, as measured by the ratio of placenta weight to pup weight (Gillis et al., 2016), was significantly improved in the DSS + Ex group relative to the DSS group (Figure 5c). Placenta efficiency in the DSS + Ex group significantly correlated with wheel rotations (*p* = 0.048) (Figure 5d).

#### 3.1.5 Proteinuria

**FIGURE 6:**
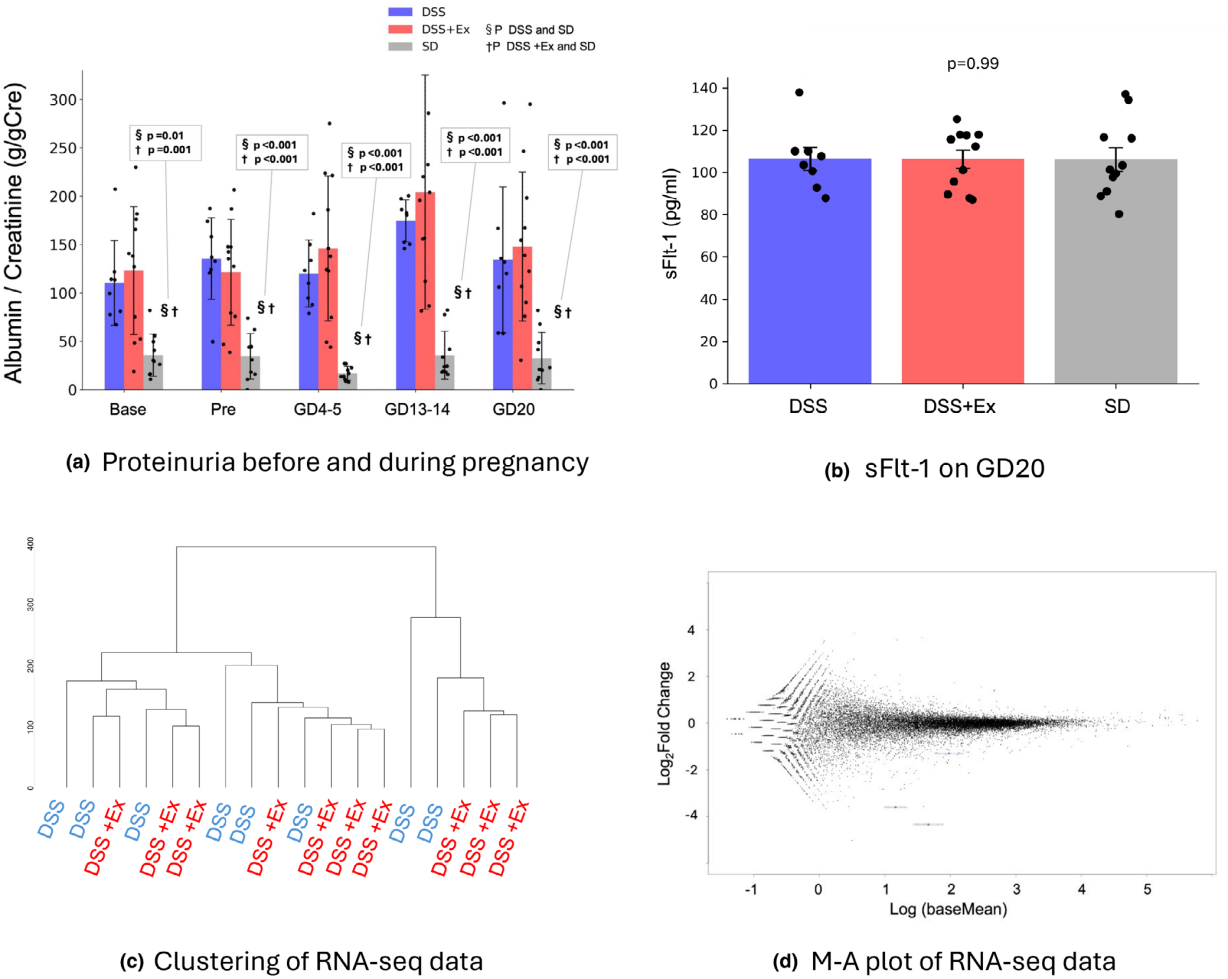
Effects of exercise on proteinuria and placental RNA sequencing in Experiment 1. (a) Proteinuria before and during pregnancy. Significant differences were observed in the SD group compared to DSS and DSS + Ex across time points (*p* < 0.001). (b) Soluble Flt‐1 levels in late gestation showed no significant differences (*p* = 0.99). These parameters were compared in the DSS (*n* = 11), DSS + Ex (*n* = 11), and SD (*n* = 10) groups. Data were expressed as mean ± SD and analyzed by one‐way ANOVA. (c, d) Clustering and M‐A plot of placental RNA sequencing in DSS (*n* = 8) and DSS + Ex (*n* = 10). Several specimens could not be tested due to improper sample collection or purification.

The non‐exercise and exercise DSS groups had more urinary protein before pregnancy than the SD group, with urinary protein increasing at the peak of mid‐pregnancy. Non‐exercise and exercise DSS rats showed no significant differences (Figure 6a).

#### 3.1.6 Angiogenic factor

In late pregnancy, the antiangiogenic factor sFlt‐1 did not differ between the three groups (Figure 6b).

#### 3.1.7 Placenta RNA sequencing

Clustering analysis was performed on the placental RNA‐seq data to discern patterns of gene expression among the samples. However, the resulting clusters showed no discernible or consistent patterns, suggesting a lack of distinct grouping or segregation of samples based on their gene expression profiles (Figure 6c). The MA plot was generated to visualize differential gene expression. Most genes clustered around the zero line on the y‐axis, indicating that the two groups lacked significant differential expression (Figure 6d).

### 3.2 Experiment 2

#### 3.2.1 Gene expression in RVLM and NTS

The RVLM and NTS reference genes were each investigated by RefFinder (Xie et al., 2023), and both were non‐POU domain‐containing octamer‐binding (NONO).

**FIGURE 7:**
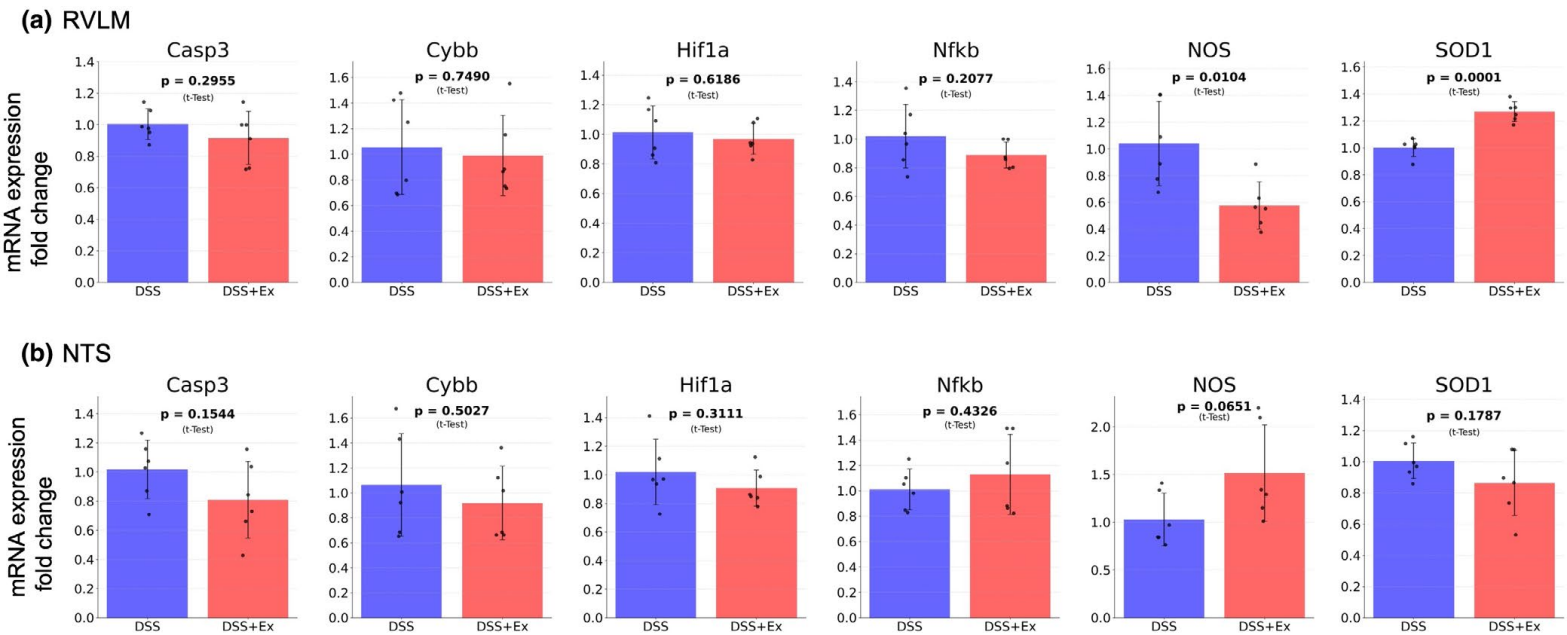
Effects of exercise on gene expression profiles in the brain (Experiment 2). (a) Gene expression analysis in the RVLM shows significant downregulation of NOS and upregulation of SOD in DSS + EX (*n* = 6) compared with DSS (*n* = 6). (b) Gene expression analysis in the NTS shows no significant differences between DSS + EX and DSS. Data were expressed as mean ± SD and analyzed by *t*‐test for parametric data.

Gene expression analyses in the RVLM revealed significant downregulation of neuronal nitric oxide synthase (NOS) (fold change relative to DSS = 0.56 ± 0.17, *p* = 0.010) and upregulation of Superoxide dismutase (SOD) (fold change relative to DSS = 1.26 ± 0.04, *p* = 0.0001) in DSS + EX compared with DSS (Figure 7a). Gene expression analysis in the NTS showed no significant difference between DSS and DSS + Ex (Figure 7b).

#### 3.2.2 Gene expression in the placenta

**FIGURE 8:**
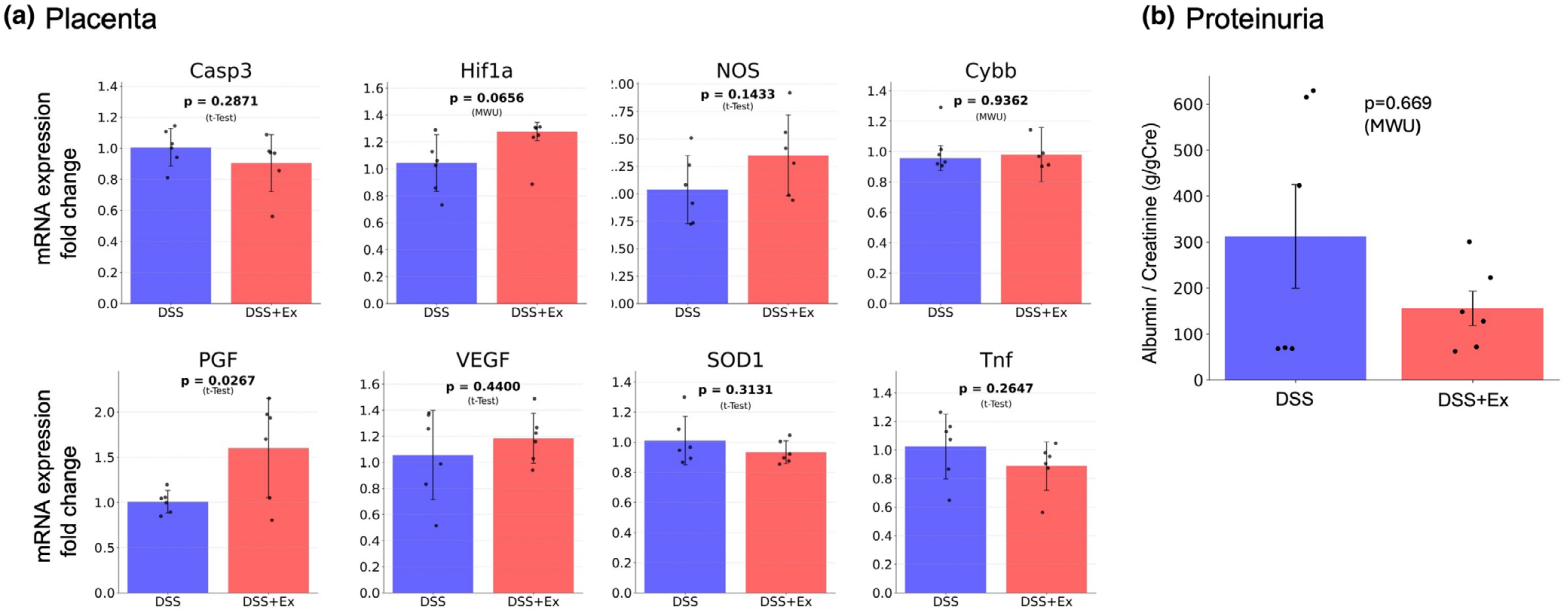
Effects of exercise on placental gene expression and proteinuria in Experiment 2. (a) Gene expression analysis in the placenta showing significant upregulation of PGF in DSS + EX (*n* = 6) relative to DSS (*n* = 6). (b) Proteinuria at GD8 showing no significant difference between DSS and DSS + Ex. Data were expressed as mean ± SD and analyzed by t‐test for parametric data and Mann–Whitney U (MWU) test for nonparametric data.

The placenta reference gene was TATA box binding protein (Tbp). Gene expression analysis in the placenta revealed significant upregulation of placenta growth factor (PGF) (fold change relative to DSS = 1.51 ± 0.24, *p* = 0.027) in DSS + EX compared with DSS, and other genes showed no significant differences (Figure 8a).

#### 3.2.3 Proteinuria

Proteinuria at GD8 showed no significant difference between DSS and DSS + Ex (Figure 8b).

## 4 DISCUSSION

We uncovered several interesting findings in this study. First, DSS rats that experienced elevated blood pressure on early pregnancy showed a reduction in blood pressure with exercise before and during pregnancy, compared to sedentary DSS rats. This reduction was associated with higher fetal weight and improved placental efficiency following daily exercise initiated prior to pregnancy. Second, we found that daily exercise in DSS rats changed gene expression in the RVLM and BRG, both of which appear to contribute to reducing elevated blood pressure levels. Third, improvements in PGF gene expression in the placenta on early pregnancy and fetal development were found after exercise. Conversely, no improvement was observed in urinary protein levels; notably, daily exercise did not exacerbate levels of sFlt‐1.

Our findings indicate that exercise in DSS is effective in reducing blood pressure, which increases during pregnancy. Although we hypothesized that DSS rats would have elevated blood pressure in mid and late gestation, as in previous studies, our study shows that the blood pressure of Dahl rats decreased after mid‐gestation. The variations in phenotype, influenced by differences in rearing and breeding environments, may have contributed to this disparity. Notably, Margaret et al. suggested that the DSS female rat phenotype was environment‐specific (Zimmerman & Lindsey, 2016). Although the animal model differed from what was expected based on previous studies, the significant differences observed on early pregnancy highlight the importance of exercise in managing elevated blood pressure and provide valuable insights into its effects.

Studies have explored the effects of exercise on hypertension during pregnancy in model rats through artificial intervention, including surgical and genetic manipulation (Falcao et al., 2010; Gilbert et al., 2012). Since the mode of onset of these conditions differs from that of humans, researchers anticipated observing similar effects in a model in which the condition arises spontaneously (Gatford et al., 2020). Therefore, our study is the first to demonstrate the effect of exercise in a spontaneous model of elevated blood pressure in pregnancy.

We observed that blood pressure was significantly lower in DSS rats with exercise compared to sedentary DSS rats, particularly on early pregnancy. Although repeated‐measures ANOVA did not detect a significant group difference in spontaneous baroreceptor reflex gain (sBRG) over the full course of pregnancy, we did find a significant correlation between sBRG and blood pressure at early gestation (*p* = 0.04). This finding suggests that improved baroreflex function may contribute, at least in part, to the observed blood pressure reduction on early pregnancy. This finding aligns with clinical studies showing that pregnant women with preeclampsia often exhibit reduced baroreflex sensitivity (Molino et al., 1999; Silver et al., 2001). Furthermore, exercise has been reported to improve BRG in other forms of hypertension as well (Laterza et al., 2007; Waki et al., 2003). However, because correlation alone cannot establish causation, further studies are needed to clarify the precise role of baroreflex in mediating blood pressure changes under these conditions.

In the present study, exercise training improved sBRG without significantly altering low‐frequency (LF), high‐frequency (HF), or the LF/HF ratio—measures commonly used in heart rate variability (HRV) analysis. A likely explanation is that sBRG reflects the acute autonomic response to blood pressure fluctuations, while LF and HF indices mainly represent resting sympathetic and parasympathetic modulation (Malik, 1996; Parati et al., 2000).

The cardiovascular control regions of the RVLM and NTS are crucial for generating and maintaining sympathetic activity and serve as essential components of the central baroreflex pathways (Granata et al., 1983). Regular exercise improves hypertension and changes gene expression in both the RVLM and NTS in non‐pregnant hypertensive rats (Farah et al., 2021; Li et al., 2018). Neuronal nitric oxide synthase (nNOS) is an element involved in inflammation (Baig et al., 2015), whereas superoxide dismutase (SOD) is an element of the antioxidant defense system (Deponte, 2013). In the RVLM, our findings of decreased nNOS and increased SOD gene expression were consistent with the changes reported in previous studies investigating the effects of exercise in non‐pregnant hypertensive rats (Farah et al., 2021; Raquel Hde et al., 2016). In contrast, the NTS did not show any clear significant differences, a finding that partly differs from some earlier reports. Additionally, there is ongoing controversy over whether endogenous nitric oxide (NO) increases or decreases sympathetic nerve activity (SNA) (Kumagai et al., 2012). Some studies suggest that NO reduces RVLM neuron activity and sympathetic outflow (Kishi et al., 2002; Kumagai et al., 1997), whereas other findings indicate that NO derived from inducible nNOS in the RVLM may increase blood pressure (Kimura et al., 2005). Further research is needed to elucidate these mechanisms and to clarify the precise role of NO in the RVLM in regulating SNA and blood pressure.

Interestingly, we observed opposing changes in nNOS expression between the RVLM and NTS—a decrease in the RVLM and a borderline increase in the NTS (*p* = 0.065, *n* = 6). Although not statistically significant, the NTS finding may still be biologically relevant. To quantify this effect, we calculated Cohen's d, which was −1.20, indicating a large effect size (Cohen, 1992). This suggests that the observed difference is substantial despite the *p* value. Because NTS‐derived nNOS is generally indicated to enhance baroreflex sensitivity (Lin et al., 2012; Murphy et al., 2013), our results in the NTS are consistent with that notion. Notably, when the NTS barosensitive neurons are activated, they suppress the RVLM sympathetic premotor neurons through GABAergic inhibitory neurons in the caudal ventrolateral medulla (CVLM), ultimately leading to a decrease in sympathetic outflow and a reduction in blood pressure. Conversely, when the NTS is inhibited, the RVLM becomes activated, resulting in increased sympathetic activity and an elevation in blood pressure (Waki, 2012). From this perspective, the fact that exercise intervention led to an opposite pattern of nNOS gene expression in the NTS and RVLM is an interesting result, as it promotes changes in sympathetic nerve output in the same direction. However, the reason why changes in gene expression patterns occur in different brain regions remains a mystery, and further investigation is needed to address this issue in the future.

Exercise improves spontaneous fetal growth restriction. Several factors can cause fetal growth restriction. Its occurrence in PE may result from abnormal placental blood flow (Chappell et al., 2021). Exercise can improve abnormal blood flow in the placenta, which may be related to reduced oxidative stress (Clapp 2003; Gilbert et al., 2012). Our comprehensive analysis of placentas from non‐exercise and exercise DSS rats in the last trimester of pregnancy revealed no significant differences. However, a significant increase in the PGF gene in the placenta on early pregnancy was observed in the exercise group. PGF gene expression is closely associated with placental perfusion in early pregnancy (Plaisier et al., 2007; Tayade et al., 2007). PGF is mainly expressed in placental trophoblasts, and its expression decreases in PE, which is associated with placental hypoxia (Ahmed et al., 2000; Makris et al., 2016). These findings may indicate that exercise improves placental formation during early pregnancy, resulting in fetal growth restriction.

While no consensus exists on the optimal amount of exercise, evidence from animal studies suggests that treadmill exercises can be stressful, and human studies indicate that labor‐intensive activities may lead to pregnancy complications. Therefore, we believe that recreational exercise, as employed in this experiment, is preferable (Cai et al., 2019; Moraska et al., 2000; Sorensen et al., 2003). Our study found a correlation between the number of wheel rotations and placental efficiency, suggesting that for humans, longer periods of low‐stress exercise may be beneficial for fetal development.

One of the limitations of this study is that the animal phenotypes were not fully representative. Previous studies have reported that DSS rats are a useful model for preeclampsia (PE) even without salt loading (Gillis et al., 2015). However, in our laboratory, neither an increase in blood pressure during the mid‐to‐late gestation of pregnancy nor an elevation in sFlt‐1 levels was observed. These differences may be due to phenotypic variation, as the baseline blood pressure of DSS rats prior to pregnancy differs. In studies where DSS rats (SS/jr) exhibited imposed PE, blood pressure was significantly higher than in SD rats even without salt loading (Gillis et al., 2015; Maeda et al., 2021). In contrast, DSS rats from Japanese strains (DIS/Eis) seem to have lower baseline blood pressure unless salt loading is applied (Ogawa et al., 2020; Yoshimoto et al., 2019). We consider these variations in phenotype and environment to be one of the reasons for the distinct phenotype observed in our rats (Zimmerman & Lindsey, 2016). On the other hand, we did observe elevated blood pressure in early pregnancy and fetal growth restriction, and it is noteworthy that the beneficial effects of exercise were demonstrated for these conditions. Another limitation of our study is the absence of mRNA expression analysis in the brain and placenta during the last trimester of pregnancy in Experiment 1, as assessed by PCR. This is because the RVLM was not collected in Experiment 1, and for the placenta, most mRNA was used in a comprehensive analysis. For placental mRNA in the last trimester, our analysis of differentially expressed genes through RNA sequencing confirmed no significant differences in PGF, suggesting that no changes occur in the last trimester. In addition, while using SD rats as controls in Experiment 2 might have been ideal, this experiment focused on exploring the mechanisms behind the exercise effects. Considering animal welfare, we believe it was appropriate not to use SD rats.

In conclusion, this study provides new information on the effects of exercise on blood pressure and fetal growth during pregnancy. Our results suggest that exercise improves elevated blood pressure during pregnancy by altering gene expression in the brain and enhancing baroreceptor reflexes. Additionally, exercise in early pregnancy may effectively improve fetal growth restriction.

## AUTHOR CONTRIBUTIONS

T.K., A.I., and H.W. conceived and designed the research; T.K., L.P., and K.Y. performed the experiments; T.K. and M.K. analyzed the data; T.K., K.Y., A.I., and H.W. interpreted the results of the experiments; T.K. prepared the figures; T.K. drafted the manuscript; K.Y., A.I., and H.W. edited and revised the manuscript.

## FUNDING INFORMATION

This work was financially supported by the Japan Society for the Promotion of Science, Grants‐in‐Aid for Scientific Research (22K09605 to A. I.); the Project Research of the Japanese Center for Research on Women in Sport, Juntendo University (to T. K. and H. W.).

## CONFLICT OF INTEREST STATEMENT

No conflicts of interest, financial or otherwise, are declared by the authors.

## ETHICS STATEMENT

This study was conducted in accordance with the guidelines of the Japan Physiological Society and approved by the Animal Experiment Ethics Committee of Juntendo University (Approval numbers: S22; 2021–16, 2022–19, 2023–19). All animal procedures were performed following the National Institutes of Health (NIH) Guide for the Care and Use of Laboratory Animals (8th edition, 2011).

## Data Availability

Data will be made available upon reasonable request.
